# Renal damage and old age: risk factors for thrombosis in patients with ANCA-associated vasculitis

**DOI:** 10.1186/s12959-024-00593-9

**Published:** 2024-03-20

**Authors:** Xin Chen, Shuo Zhang, Ruilian You, Yixin Ma, Peng Xia, Xiaoxiao Shi, Haiting Wu, Ke Zheng, Yan Qin, Xinping Tian, Limeng Chen

**Affiliations:** 1 Department of Nephrology, State Key Laboratory of Complex Severe and Rare Diseases, Peking Union Medical College Hospital, Chinese Academy of Medical Science and Peking Union Medical College, No 1, Shuaifuyuan, Wangfujing St, 100730 Beijing, China; 2 Department of Rheumatology, State Key Laboratory of Complex Severe and Rare Diseases, Peking Union Medical College Hospital, Chinese Academy of Medical Science and Peking Union Medical College, 100730 Beijing, China

**Keywords:** Antineutrophil cytoplasmic autoantibody associated vasculitis (AAV), Eosinophilic granulomatosis with polyangiitis (EGPA), Thrombosis, Mendelian randomization (MR), Renal damage

## Abstract

**Introduction:**

Thrombosis in ANCA-associated vasculitis (AAV) was prevalent and has been neglected in Chinese patients. This study tried to describe the clinical characteristics, identify the risk factors, and investigate the causal relationship between AAV and venous thromboembolism (VTE) by two-sample Mendelian randomization (MR) analysis.

**Methods:**

In this retrospective, observational study, we included all hospitalized AAV patients from Jan 2013 to Apr 2022 in Peking Union Medical College Hospital. We collected their clinical data for multivariate regression analysis to determine the risk factors for thrombosis. The nomogram was constructed by applying these risk factors to predict thrombosis in AAV patients. As for MR analysis, we selected single nucleotide polymorphisms (SNPs) related to AAV from published genome-wide association studies and extracted the outcome data containing deep vein thrombosis (DVT) and pulmonary embolism (PE) from the UK biobank.

**Results:**

1203 primary AAV patients were enrolled, and thrombosis occurred in 11.3%. Multivariate regression suggested that older than 65 years, EGPA, neurological involvement, lung involvement, significantly elevated serum creatinine (> 500µmol/L), and elevated D-dimer were associated with thrombosis in AAV patients. The model demonstrated satisfied discrimination with an AUC of 0.769 (95% CI, 0.726–0.812). MR analysis showed that EGPA could increase the risk of developing DVT and PE (OR = 1.0038, 95%CI = 1.0035–1.0041, *P* = 0.009).

**Conclusion:**

Thrombosis was not rare in Chinese patients with AAV. Renal damage and old age emerged as critical risk factors for thrombosis. EGPA might have a potential causal relationship with DVT and PE.

**Supplementary Information:**

The online version contains supplementary material available at 10.1186/s12959-024-00593-9.

## Introduction

ANCA-associated vasculitis (AAV) is a group of autoimmune diseases characterized by inflammation of blood vessels. Among the complications associated with AAV, thrombosis poses a significant threat, particularly in patients with renal damage and advanced age. The incidence of venous thromboembolism (VTE) in American patients with ANCA-associated vasculitis (AAV) was more than three times higher than in non-AAV subjects, especially for deep vein thrombosis (DVT) [1]. The prospective study in the Netherlands showed that the VTE incidence of AAV patients was 1.8 per 100 person-years [2]. Most studies included males, elevated CRP, and deteriorating renal function as risk factors for thrombosis and confirmed that age played a crucial role in thrombosis [3–5]. However, because of the racial differences in AAV classification, MPO-ANCA positive AAV was prevalent in Asian countries [6] with unknown thrombosis, neither the risk factors. It’s critical to explore the intricate relationship between renal damage, old age, and the heightened risk of thrombosis in the Asian population diagnosed with ANCA-associated vasculitis.

In addition, the causal relationship between AAV and VTE events remained unclear. Animal studies indicated that endothelial injury played a critical role, contributed by neutrophil activation, which results in the formation of neutrophil extracellular traps (NETs) and the overexpression of tissue factors leading to thrombosis [7]. Illustrating the associated causal relationship through clinical research is a pretty big challenge. Mendelian randomization (MR) is an emerging research method that can simulate randomized controlled trials using genetic variants as instrumental variables. As a natural randomization method, the gene allocated randomly at conception minimizes the effects of confounders, and MR is widely applied to estimate the causal effect of exposure on outcome [8]. Therefore, MR analysis could be a way to figure out the connection between AAV and thrombosis.

This study mainly focused on exploring the clinical characteristics of AAV patients with thrombosis, finding the risk factors in the retrospective cohort in a single-center hospital in China, and developing a nomogram model for clinical usage. Then, we also applied two-sample MR to investigate the causal relationship between the subtype of AAV and thrombosis.

## Method

### Participants and data collection

**Fig. 1:**
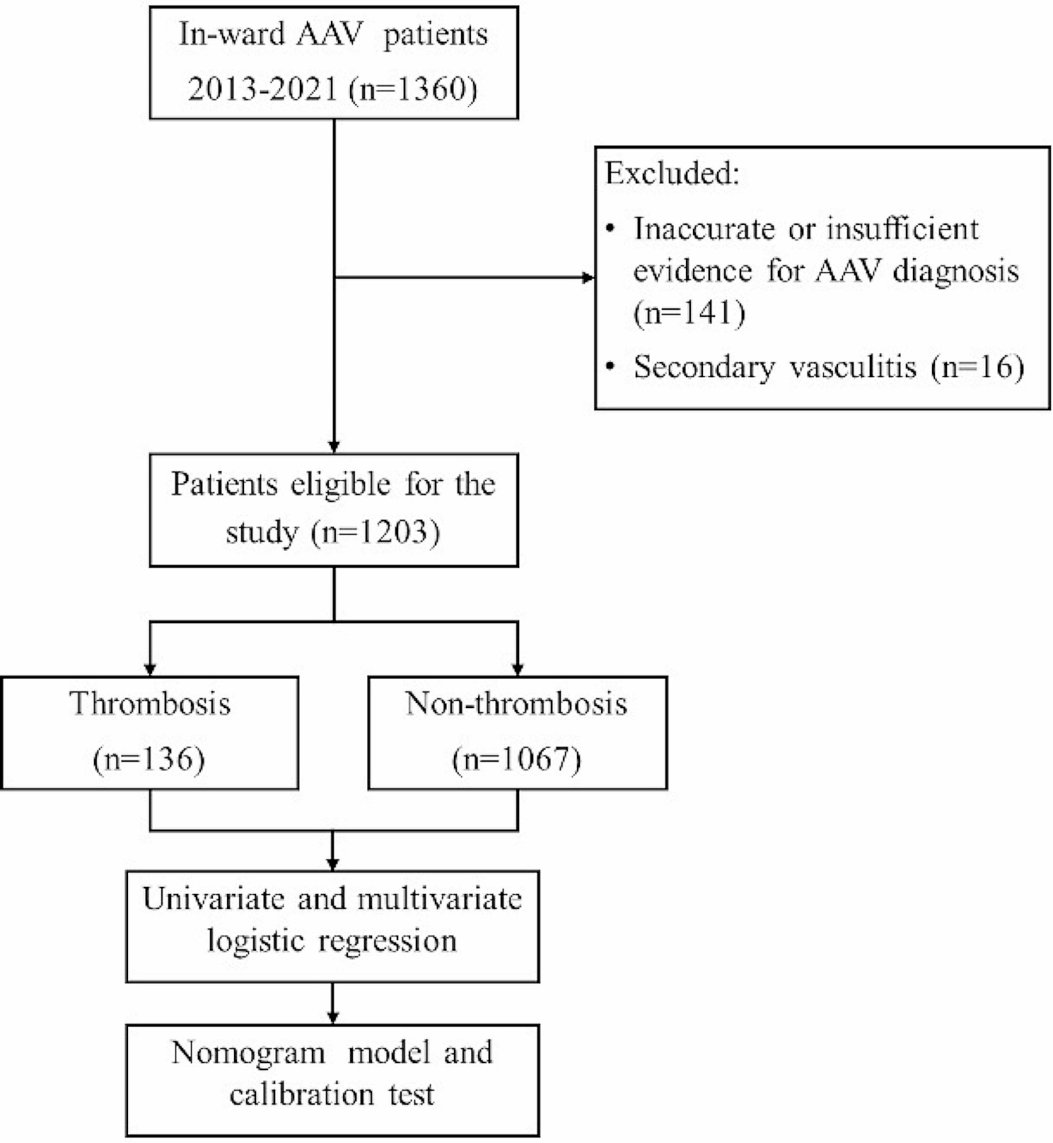
The flow chart of the study. *AAV, ANCA-associated vasculitis

In this retrospective, observational study, all patients with AAV hospitalized in Peking Union Medical College Hospital from 2013 Jan. to 2022 Apr. were included, excluding the insufficient evidence for AAV diagnosis or secondary vasculitis (Fig. 1). Data were collected from an electronic medical records database, and lab examinations were taken on admission day when diagnosed as AAV. Clinical characteristics included age, sex, symptoms, system involvement, comorbidities, AAV classifications, lab results, treatments, and short-term outcomes at the hospital discharge. The Peking Union Medical College Hospital Ethical Committee approved this study (No. JS 3527).

### Definitions

AAV was diagnosed and classified according to the 2012 revised International Chapel Hill Consensus Conference [9]. The exclusion criteria included mimicking vasculitis and secondary vasculitis. We record all diagnosed thrombosis using a multifaceted approach, including clinical assessment, pre-test probability evaluation, and objective diagnostic testing [10]. VTE is a condition that occurs when a blood clot forms in a vein, including DVT and pulmonary embolism (PE) [11]. Arterial thromboembolism (ATE) includes myocardial infarction, ischemic/unspecified stroke, and peripheral arterial occlusion [12]. The lung involvement in AAV included necrotizing granulomatous inflammation, tracheobronchial inflammation, pulmonary capillarity, interstitial lung disease (ILD), and asthma with their clinical, radiological, and therapeutic characteristics. The neurological involvement included peripheral and central nervous system manifestation, including sensory peripheral neuropathy, mononeuritis multiplex, and stroke. End-stage renal disease (ESRD) was defined as an estimated glomerular filtration rate (eGFR) less than 15 ml/min/1.73m [2] and necessary for long-term dialysis [13]. Hypoalbuminemia was a serum albumin level of less than 30 g/L. Serum creatinine was classified based on the Birmingham Vasculitis Activity Score (version 3) [14]. The lab results were according to hospital settings of normal ranges, such as D-dimer (< 0.55 mg/L), erythrocyte sedimentation rate (ESR < 15 mm/h for male, < 20 mm/h for female), C-Reactive Protein (CRP, <8 mg/L), complement 3 (C3 0.73-1.46 g/L), and complement 4 (C4 0.10-0.40 g/L).

### MR study design

**Fig. 2:**
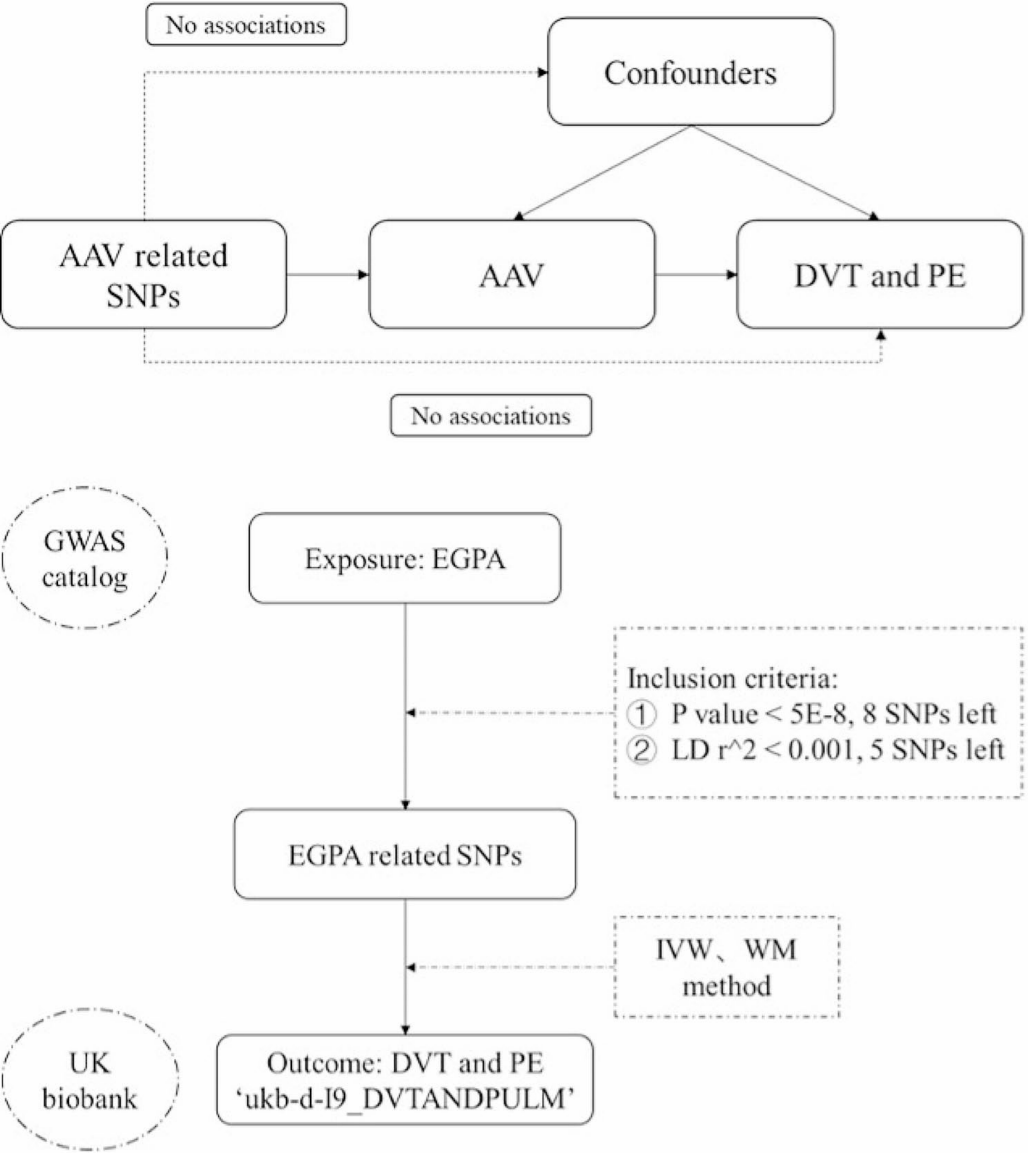
Directed acyclic graph composed of the genetic instrument (AAV-related SNPs), exposure (AAV), and outcome (DVT and PE). *EPGA, eosinophilic granulomatosis with polyangiitis; SNPs, Single nucleotide polymorphisms; DVT, deep venous thrombosis; PE, pulmonary embolism; LD, linkage disequilibrium; IVW, Inverse variance weighted; WM, weighted median

MR analysis is based on three assumptions, including that (1) the instrumental variable (IV) is closely associated with the exposure; (2) IV is not associated with any potential confounders; and (3) IV can only influence the outcome via the exposure. We constructed a directed acyclic graph by using genetic instruments (AAV-related SNPs), exposure (AAV), and outcomes (DVT and PE) (Fig. 2).

### MR data selection

Interpreted from the logistic regression of the risk factors for thrombosis in AAV patients in the retrospective cohort, we conducted MR analysis on the causal relationship between EGPA and thrombosis. First, we extracted the SNP associated with EGPA from the published article about EGPA in Nature Communication 2019 [15]. The study was a genome-wide association study including 676 EGPA cases and 6809 controls in the European population. We selected IVs with *P* ≤ 5 × 10^−8, minor allele frequency (MAF) > 0.01, and low linkage disequilibrium (LD) (r2 < 0.1). Finally, five SNPs (rs17212014, rs72689399, rs1837253, rs72946301, rs78478398) were valid for further MR analysis of EGPA. The polymorphisms of five SNPs were tested and proved to be independent of confounding factors.

The outcome data (DVT and PE) came from the UK biobank (http://www.nealelab.is/uk-biobank). First, we searched the traits ‘thrombosis’, ‘deep venous thrombosis’, and ‘pulmonary embolism’ as keywords filtered by the European population in the database. Then, we chose the most considerable associated data as the outcome (ukb-d-I9_DVTANDPULM). It was a European dataset considering 4,319 cases, 356,875 controls, and 11,783,033 SNPs.

### Data analyses

The statistical analyses of the observational study were by SPSS statistics for Windows (Version 20.0. IBM Corp, Armonk, NY) and R statistical software (V.4.0.0). Categorical variables were expressed as absolute (n) and relative (%) frequency by the Pearson 2-test. Continuous variables were expressed as mean ± standard deviation (SD) and analyzed by unpaired t-tests. Continuous variables that violated the normality assumption were expressed as median and 25th to 75th percentiles and analyzed by a Mann–Whitney U-test. After performing baseline analysis, we selected risk factors with statistical significance and clinical relevance in univariate regression analysis. Then, the factors with statistical significance in the univariate regression analysis were included in the multivariate logistic regression, and the odds ratios and 95% CIs were calculated. Variables with statistical significance based on the multivariate analysis were contained in the nomogram. The area under the receiver operating curve (AUC) was calculated to assess the apparent performance of the nomogram [16]. Meanwhile, the calibration plot and Hosmer-Lemeshow goodness-of-fit (HL) test were used to evaluate the accuracy by comparing the nomogram. Decision curve analysis (DCA) was performed to assess the clinical usefulness of the nomogram [17]. A *p*-value < 0.05 indicates statistically significant differences.

The MR analysis used R 4.0.0 software of packages ‘Two Sample MR’ and ‘MR-PRESSO’. To ensure that the effect allele of IVs in exposure and outcome in different databases correspond to the same allele, we inferred the forward strand alleles using frequency information to harmonize the data, discarded ambiguous IVs or not inferable palindromic ones. We performed MR analysis through robust analytical methods, including weighted median (WM) and inverse variance weighted (IVW), Cochran’s Q statistic to assess heterogeneity between individual genetic variants in the IVW method, and scatter plots and leave-one-out to evaluate the robustness of the findings. We adopted Mendelian Randomization Pleiotropy Residual Sum and Outlier (MR-PRESSO) to detect and correct potential outliers.

## Result

### Clinical characteristics of AAV patients with thrombosis

A total of 1203 patients were included in the study, and males accounted for 52%. The mean age was 55 ± 17 years, and age > 65 accounted for 36%. MPA, GPA, and EGPA accounted for 50.0%, 29.7%, and 15.8%, respectively. Among all the patients, 136 patients (11.3%) acquired thrombosis, with VTE (10.3%), ATE (0.93%), DVT (8.5%), and PE (1.8%). AAV patients with thrombosis had a higher percentage of elderly patients (60 ± 14 vs. 54 ± 17 years, *P* < 0.001), more smoking (37.5% vs. 31%, *P* = 0.003) and more chronic kidney disease (CKD, 22.8 vs. 15.6%, *P* = 0.032). AAV patients with thrombosis had hypoalbuminemia (33.3% vs. 24.8%, *P* = 0.035), renal impairment with more proportion of elevated serum creatinine (Scr > 500µmol/L, 18.2% vs. 7.3%, *P* < 0.001) and elevated D-dimer (3.0 vs. 1.2 mg/L, *P* < 0.001). There was no statistical difference in the levels of ANCA, inflammatory indicators (CRP and ESR), and complements. The complications were more evident in patients with thrombosis, including a higher percentage of infection (36% vs. 25.7%, *P* = 0.010) and anemia (41.2% vs. 30.4%, *P* = 0.011). After immunosuppressive treatments, the all-cause death rates were of no statistical differences during in-hospital, but AAV with thrombosis with prolonged hospitalization (29.6 ± 17.1 vs. 22.6 ± 13.2 days, *P* < 0.001). (Table 1)

**Table 1:** Baseline clinical characteristics of patients with or without thrombotic events in AAV patients

Variables	Total (*n* = 1203)	Thrombosis (*n* = 136)	Non-thrombosis (*n* = 1067)	*P* value
Age	55 ± 17	60 ± 14	54 ± 17	< 0.001
Gender (Male)	625 (52.0%)	71 (52.2%)	554 (51.9%)	0.95
Smoking	382 (31.8%)	51 (37.5%)	331 (31.0%)	0.003
System involvement				
Kidney	785 (65.3%)	91 (66.9%)	694 (65.0%)	0.666
Lung	660 (54.9%)	95 (69.9%)	565 (53%)	< 0.001
Neurological	216 (18.0%)	41 (30.1%)	175 (16.4%)	< 0.001
ENT	322 (26.8%)	41 (30.1%)	281 (26.3%)	0.344
Commodities				
Hypertension	527 (43.8%)	72 (52.9%)	455 (42.6%)	0.023
Diabetes	341 (28.4%)	32 (23.5%)	309 (29%)	0.186
CHD	49 (4.1%)	7 (5.1%)	42 (3.9%)	0.501
CKD	197 (16.4%)	31 (22.8%)	166 (15.6%)	0.032
ESRD	151 (12.6%)	32 (23.5%)	119 (11.2%)	< 0.001
COPD	40 (3.3%)	6 (4.4%)	34 (3.2%)	0.453
Stroke	93 (7.7%)	16 (11.8%)	77 (7.2%)	0.061
Malignancy	74 (6.15%)	8 (5.88%)	66 (6.19%)	0.890
AAV classification				0.003
MPA	602 (50.0%)	63 (46.3%)	539 (50.5%)	
GPA	357 (29.7%)	31 (22.8%)	326 (30.6%)	
EGPA	190 (15.8%)	37 (27.2%)	153 (14.3%)	
ANCA				
p-ANCA	493(41.0%)	47(34.6%)	446(41.8%)	0.106
c-ANCA	217(18.0%)	24(17.6%)	193(18.1%)	0.9
MPO	197(16.4%)	21(15.4%)	176(16.5%)	0.754
PR3	464(38.6%)	50(36.8%)	414(38.8%)	0.646
Hypoalbuminemia	301 (25.8%)	44 (33.3%)	257 (24.8%)	0.035
EOS (×10^9/L)	0.20 (0.02-1.00)	0.14 (0.01–0.59)	0.20 (0.02-1.00)	0.113
Scr (µmol/L)				< 0.001
< 124	720 (61.7%)	68 (51.5%)	652 (63.1%)	
125–249	187 (16.0%)	20 (15.2%)	167 (16.2%)	
249–499	160 (13.7%)	20 (15.2%)	140 (13.5%)	
> 500	99 (8.5%)	24 (18.2%)	75 (7.3%)	
D-dimer (mg/L)	1.4 (0.6–3.5)	3.0 (1.3–17.2)	1.2 (0.5–3.1)	< 0.001
Elevated ESR	766 (72.8%)	77 (66.4%)	689 (73.6%)	0.099
Elevated CRP	633 (57.7%)	76 (60.8%)	557 (57.2%)	0.449
Decreased C3	110 (13.1%)	16 (16.7%)	94 (12.7%)	0.273
Decreased C4	60 (7.1%)	10 (10.4%)	50 (6.7%)	0.176
Complications				
Infection	323 (26.9%)	49 (36%)	274 (25.7%)	0.01
Anemia	380 (31.6%)	56 (41.2%)	324 (30.4%)	0.011
Treatment				
GC po.	1162 (96.6%)	133(97.8%)	1029(96.4%)	0.412
GC pulse.	298 (24.8%)	38 (27.9%)	260 (24.4%)	0.363
CTX	868 (72.2%)	103 (75.7%)	765 (71.7%)	0.322
RTX	75 (6.2%)	6 (4.4%)	69 (6.5%)	0.351
PE	39 (3.2%)	7 (5.1%)	32 (3%)	0.183
Dialysis	113 (9.39)	30 (22.06)	83 (7.78)	< 0.001
Death	63 (5.2%)	7 (5.2%)	56 (5.3%)	0.96
Length of stay (days)	23.3 ± 13.8	29.6 ± 17.1	22.6 ± 13.2	< 0.001

*AAV, ANCA-associated vasculitis; MPA, microscopic polyangiitis; GPA, granulomatosis with polyangiitis; EGPA, eosinophilic granulomatosis with polyangiitis; ENT, Ear, nose, and throat; CHD, chronic heart disease; CKD, chronic kidney disease; COPD, chronic obstructive pulmonary disease; eGFR, estimated glomerular filtration rate; FBG, fibrinogen; ESR, erythrocyte sedimentation rate; CRP, C-Reactive Protein; GC pulse., intravenous pulse glucocorticoid; GC po., glucocorticoid peros; CTX, cyclophosphamide; RTX, rituximab; PE, plasma exchange

MPA, GPA, and EGPA thrombosis were 10.5%, 8.7%, and 19.5% (*P* = 0.003). The clinical characteristics and the thrombosis rate in different AAV classifications were described in sTable 1–4. All MPA, GPA, and EGPA patients with thrombosis were older, acquired elevated D-dimer, and had prolonged hospitalization. MPA patients with thrombosis acquired worse renal function with more CKD, ESRD, elevated Scr, and more anemia. GPA patients with thrombosis had more neurological involvement and infection. EGPA patients with thrombosis had more lung involvement and ESRD. EGPA had the highest rates of VTE (18.9%) and ATE (2.1%) among the three AAV (*P* = 0.026), as well as the highest rates of DVT (EGPA 16.8%, *P* < 0.001) and PE (5.3%, *P* = 0.004), without relationship to the levels of ANCA.

### Risk factors of thrombosis in AAV patients

Univariate regression suggested that age > 65, neurological or lung involvement, ESRD, and elevated D-dimer were associated with thrombosis (*P* < 0.05, Table 2). Multivariate regression suggested that age > 65 years (OR = 1.65 (1.10–2.47), *P* = 0.015), EGPA (OR = 3.50 (2.11–5.81), *P* < 0.001), neurological involvement (OR = 2.05 (1.30–3.24), *P* = 0.002), lung involvement (OR = 1.90 (1.24–2.90), *P* = 0.003), significantly elevated serum creatinine (Scr > 500µmol/L, OR = 2.78 (1.52–5.09), *P* = 0.001), and elevated D-dimer (0.55-2 mg/L, OR = 2.80 (1.41–5.56), *P* = 0.003; >2 mg/L, OR = 6.28 (3.20–11.93), *P* < 0.001), were independent risk factor of thrombosis in AAV patients.

**Table 2:** Univariate and multivariate regression analysis of thrombosis events in AAV patients

Variables	Univariate regression			Multivariate regression		
	Beta	OR (95%CI)	*P* value	aBeta	aOR (95%CI)	a*P* value
Age > 65 years	0.65	1.91 (1.33–2.74)	< 0.001	0.50	1.65 (1.10–2.47)	0.015
EGPA	0.80	2.23 (1.47–3.38)	< 0.001	1.25	3.50 (2.11–5.81)	< 0.001
Neurological	0.79	2.20 (1.47–3.28)	< 0.001	0.72	2.05 (1.30–3.24)	0.002
Lung	0.72	2.06 (1.40–3.03)	< 0.001	0.64	1.90 (1.24–2.90)	0.003
Scr > 500µmol/L	1.12	3.07 (1.82–5.18)	< 0.001	1.02	2.78 (1.52–5.09)	0.001
D-dimer						
< 0.55 mg/L		1.00 (Reference)			1.00 (Reference)	
0.55-2 mg/L	1.00	2.71 (1.48–4.96)	0.001	1.03	2.80 (1.41–5.56)	0.003
> 2 mg/L	1.80	6.06 (3.48–10.56)	< 0.001	1.82	6.18 (3.20–11.93)	< 0.001

*AAV, ANCA-associated vasculitis; EGPA, eosinophilic granulomatosis with polyangiitis; ESRD, end stage renal disease

### The nomogram and its predictive value

**Fig. 3:**
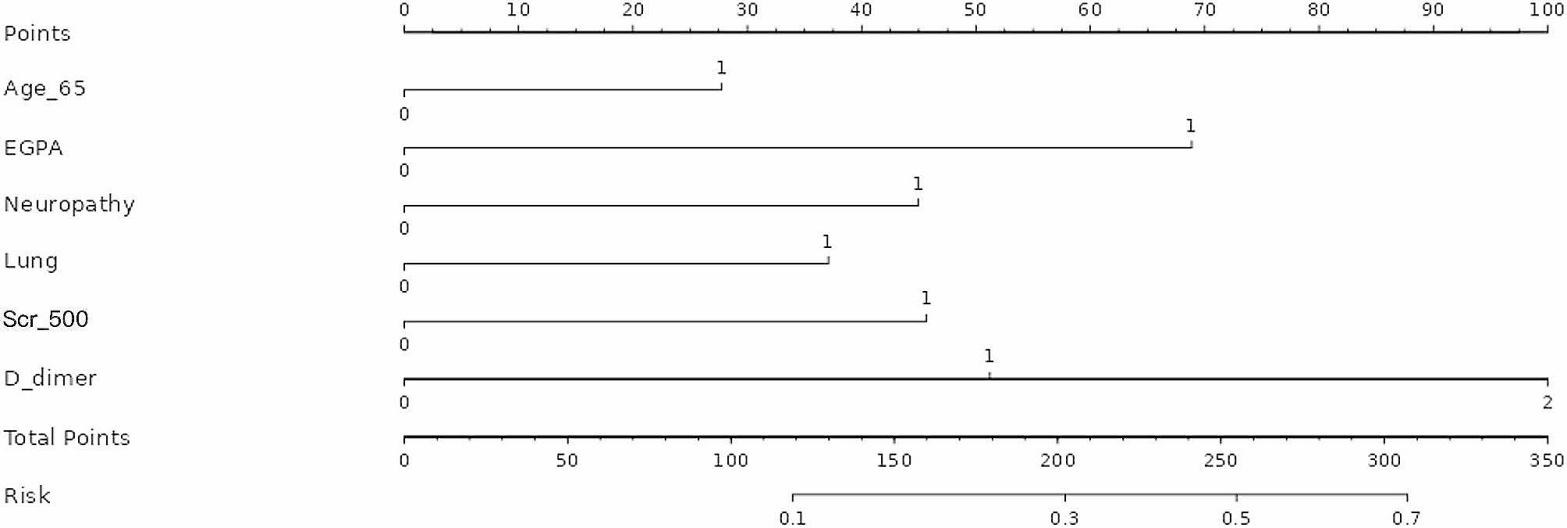
The nomogram of predicting the thrombotic events in AAV patients. *AAV, ANCA-associated vasculitis; Age_65, age > 65 years; EGPA, eosinophilic granulomatosis with polyangiitis

**Fig. 4:**
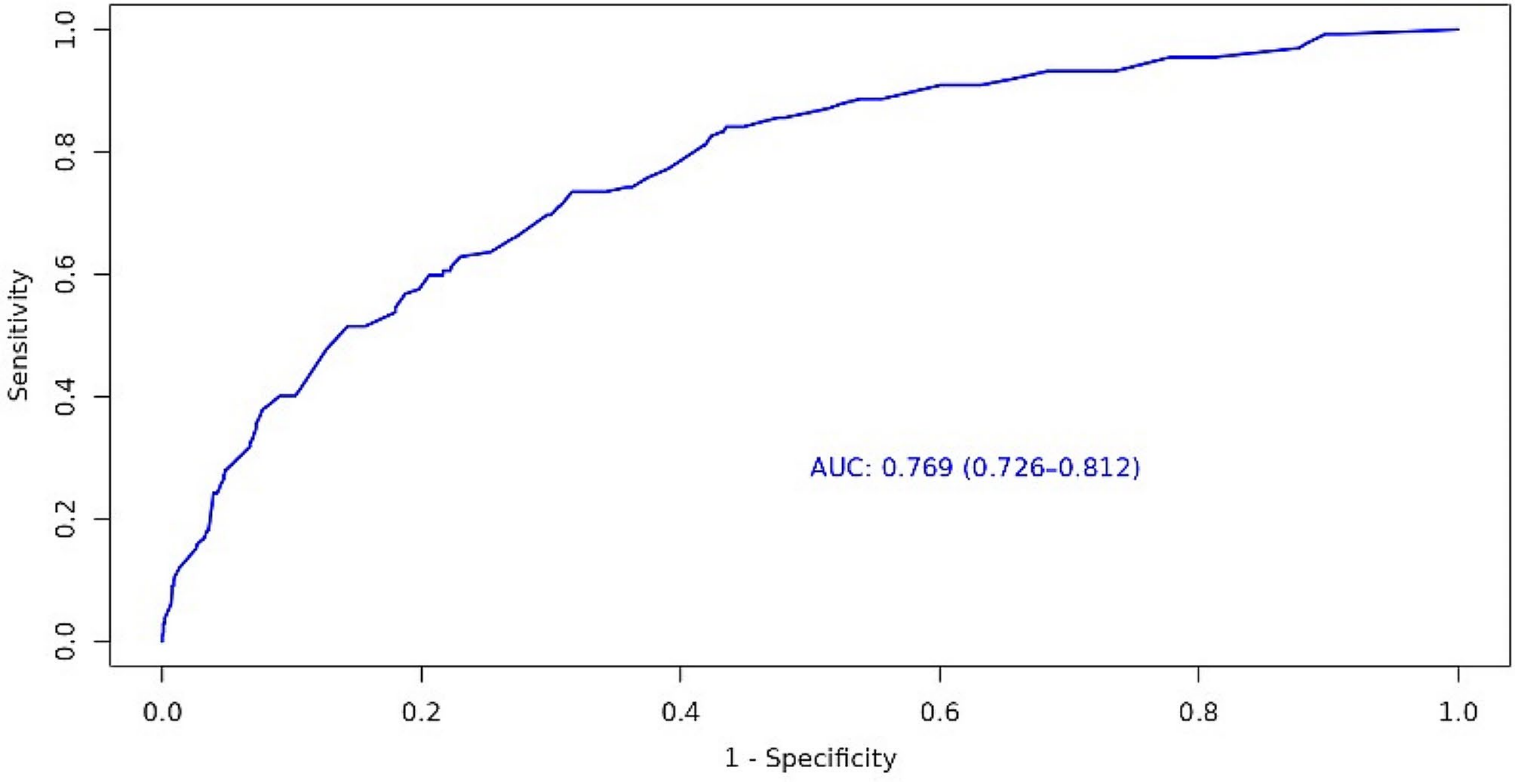
The receiver operating characteristic (ROC) curve of thrombosis events in AAV patients. *AAV, ANCA-associated vasculitis

Older than 65 years, neurological and lung involvement, significantly elevated serum creatinine, and elevated D-dimer were included in the nomogram based on the multivariate analysis (Fig. 3). The AUC for the nomogram was 0.769 (95% CI, 0.726–0.812) (Fig. 4). The calibration plot showed a good fitting degree of the nomogram (sFigure 1). The HL test of multivariable analysis also demonstrated perfect consistency (χ2 = 6.9631, *P* = 0.5406). As for clinical practice, DCA for the nomogram was conducted, and it showed a more significant net benefit than full or no treatment across a threshold probability range (sFigure 2).

### MR analysis

**Fig. 5:**
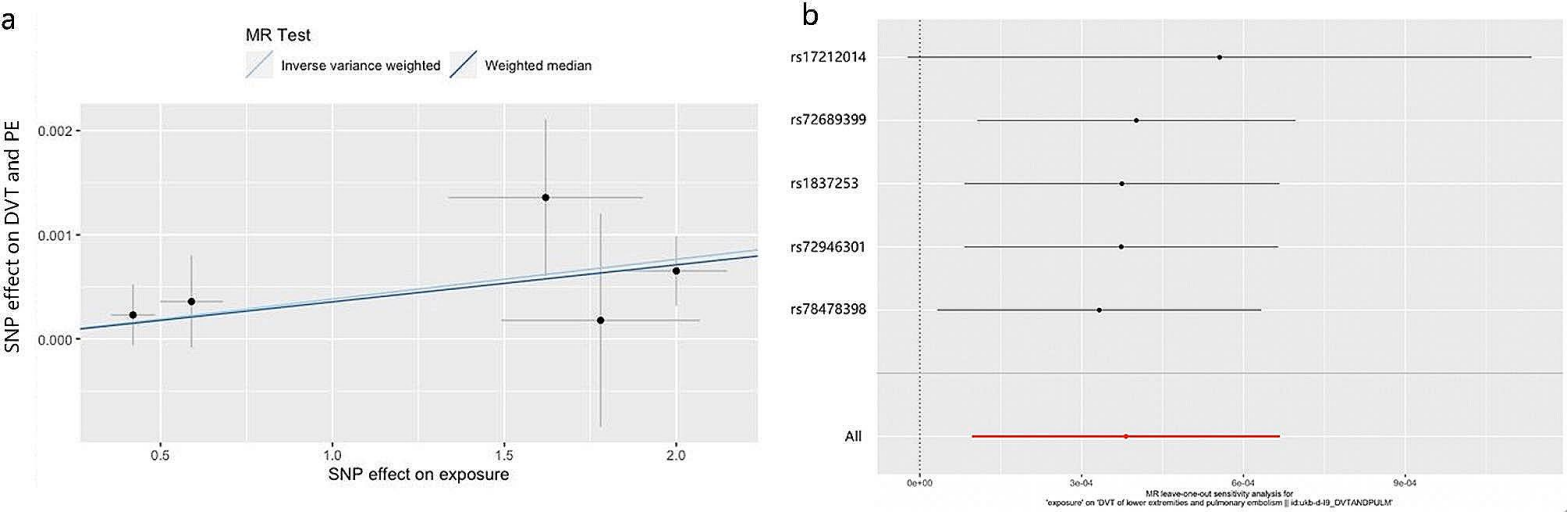
**5a**. Scatter plot of the Mendelian randomized (MR) outcome. **5b**. The associations between EGPA and thrombotic events. *EPGA, eosinophilic granulomatosis with polyangiitis; DVT, deep venous thrombosis; PE, pulmonary embolism; IVW, Inverse variance weighted; WM, weighted median

The scatter plot showed the distribution of the single SNP’s effect on the outcome (Fig. 5a). The *P*-values of heterogeneity and pleiotropy test were 0.74 and 0.69, indicating low heterogeneity and no evidence of genetic pleiotropy. The leave-one-out method suggested that the outcome was robust. MR analysis showed that EGPA could increase the risk of developing thrombosis, including DVT and PE (OR = 1.0038, 95%CI = 1.0035–1.0041, *P* = 0.009; Fig. 5b). To analyze the association between MPA or GPA and thrombosis, we extracted the MPA dataset from finngen_R8_M13_MICROPOLYANG, and the GPA dataset from finngen_R8_M13_WEGNER. No statistical significance of the relationship between MPA or GPA and thrombosis (*P* = 0.79; *P* = 0.83) was observed by the IVW method.

## Discussion

In this retrospective, observational large Asian cohort study, we illustrated that 11.3% of patients developed thrombosis out of 1203 patients with primary AAV who suffered from prolonged hospital stays. In the largest sample size of Asian patients, we concluded that age > 65 years, neurological or lung involvement, severe renal impairment, and elevated D-dimer were independent risk factors of thrombosis with satisfactory performance in the nomogram model. Furthermore, we first unveiled the causal effect between EGPA and DVT(PE) by integrating publicly available GWAS datasets.

Our results indicated that AAV patients in the Chinese population had similar incidences of VTE to Western countries, consistent with Europe (9.8%) [18], with similar VTE percentage to the data from Johns Hopkins Hospital (10.3% vs. 14%) [19]. The prospective study in the Netherlands showed that the VTE incidence of AAV patients was 1.8 per 100 person-years [2], mostly during active disease (3 months before and after diagnosis or relapse of AAV). However, the retrospective cohort in French showed no difference in VTE incidences among different AAV subtypes [3], inconsistent with our EGPA patients with the significantly highest rate of VTE (18.9%) than MPA or GPA patients.

The risk factors of thrombosis in AAV patients varied in different regions and races. Johns Hopkins Hospital reported that PR3-ANCA and hypoalbuminemia were risk factors for thrombosis in AAV patients, among which 65% were GPA [19]. In our study, among which half were MPA. Old age might increase risks for thrombosis, which was consistent with the retrospective study from France (OR = 1.29, 95%CI = 1.01–1.04, *P* = 0.002) [5]. In the previous report, the incidence of VTE was infrequent in young individuals (< 1 per 10,000 per year) but increased to about 2 per 1000 per year at the age of 65 years and continued rising in the older population [20]. The age-specific risk factors of thrombosis might be because of endothelial dysfunction and frailty exacerbated with aging [21]. In the context of AAV, older patients often present with comorbidities and physiological changes that contribute to an elevated risk of thrombosis. The aging process may affect the coagulation system, making patients more susceptible to clot formation [22].

Renal involvement is a common and severe manifestation of AAV, affecting many patients. Various studies suggested that baseline serum creatinine was associated with thrombosis [18, 23]. It might be caused by the loss of anti-thrombotic factors in the urine of patients with renal involvement and the generation of neutrophil extracellular traps (NETs) recognized as a key pathological feature in renal biopsies from patients with glomerulonephritis associated with AAV [7]. Our study suggested that the combination of severe renal damage and old age created a synergistic effect, significantly increasing the risk of thrombosis in patients with AAV. The European Vasculitis Study Group indicated other risk factors included cutaneous and gastrointestinal involvement [4]. Our study suggested that neurological and lung involvement might be associated with thrombosis. Neurologic involvement might be related to stroke events in AAV patients, which was listed as an indicator of systemic involvement in the BVAS score [14]. A nationwide cohort in Korea found that the risk of stroke was 2.3 times higher in AAV patients [24]. Lung involvement mainly manifested as interstitial lung lesions, pulmonary hemorrhage (PH), pulmonary nodules, etc. VTE and PH had a higher incidence with increasing disease activity [2]. Though rare, case reports suggested the coexistence of VTE and PH in patients with AAV [25], and VTE as an important clue for higher AAV disease activity. The mechanism might also involve severe lung involvement, and even a ventilator-assisted respiratory state led to the aggravation of the relative braking factor and caused thrombosis. More evidence should be obtained from different medical institutions.

We found a causal relationship between EGPA and VTE or PE in MR analysis. The nationwide study in Korea also found that the risks of VTE and PE were the highest in EGPA patients among AAV patients [24]. The mechanism of EGPA causing thrombosis involves multiple factors [26]. Primarily, eosinophils play a crucial role by storing and releasing tissue factors and other cationic proteins, such as major essential protein (MBP) and eosinophilic cationic protein (ECP). MBP disrupted the thrombin binding at the endothelial growth factor domain of thrombomodulin, leading to a deformed complex structure, causing a shift in thrombin’s substrate specificity from protein C to fibrinogen [27]. Additionally, ECP could prevent thrombin-dependent activation of protein C, thus blocking the anticoagulant effect of protein C [28]. Moreover, tissue factor released by eosinophil might activate factor X via factor VII [29]. The presence of ANCA also transforms the endothelium into a pro-adhesive surface, promoting thrombus formation. The combined impacts mentioned above would result in unimpeded fibrin and thrombin production, which would ultimately cause clot formation. In clinical practice, adequate and timely immunosuppression may contribute to preventing thrombosis. Patients who do not receive immunomodulatory therapy within the first two months after EGPA diagnosis are at a significantly higher risk of developing ATE or VTE compared to those treated with systemic corticosteroids (HR = 3.67(1.37–9.89), *P* = 0.010) [30]. Conversely, patients receiving immunosuppressive agents or corticosteroids as monotherapy exhibit a relatively balanced risk.

In our AAV patients, another solid indication of thrombosis is highly elevated D-dimer, which indicates both the activation of the coagulation pathway and the inflammatory state. It is associated with disease activity and inflammation in GPA patients [31] and predicted mortality and intensive care unit requirements among AAV patients in South Korea [32]. Our study suggested that the cut-off point of D-dimer (> 2 mg/L) might serve as an alerting biomarker to indicate disease activity, thrombosis, and adverse outcomes of AAV patients, which once observed in lupus patients [4].

The study has several limitations. Firstly, as a retrospective, single-center cohort study, the model’s external validity or generalized ability might need further evaluation. It’s challenging to distinguish the chronological relationship between AAV and thrombosis from the retrospective study. Secondly, in clinical practice, some thrombotic events were asymptomatic, and screening prevention was based on the physician’s judgment instead of regular tests on all patients, which may lead to underestimation of certain subclinical thrombotic events. Thirdly, the casual relationship proof from the MR analysis was from the European population’s genetic background. More prospective and Asian genome-wide association studies were needed to confirm the conclusion.

## Conclusion

Thrombosis was a common complication of Chinese AAV patients. Renal damage and old age emerged as critical risk factors for thrombosis in AAV patients. Understanding the interplay between these factors and applying nomogram models to identify potential risks were essential for clinical physicians to intervene timely and improve prognosis. Furthermore, MR analysis suggested a potential causal relationship between EGPA and the development of DVT or PE.

### Electronic supplementary material

Below is the link to the electronic supplementary material.

**Supplementary Material 1**: **sFigure 1**. Calibration curves of the predicted nomogram. The x-axis represents the predicted probability calculated by the nomogram, and the y-axis is the observed actual probability of thrombotic events in AAV patients. Results of the Hosmer-Lemeshow test demonstrate that the *P*-value of 0.541. **sFigure 2**. The decision curve analysis (DCA) of the nomogram. The horizontal line indicates no AAV patients with thrombosis, and the red diagonal line indicates AAV patients with thrombosis. The solid blue line indicates the risk nomogram. In DCA, the nomogram shows more net benefit THAN full or no treatment across a threshold probability range. **sTable 1**. The rate of thrombosis in different AAV classifications. **sTable 2**. The clinical characteristics between thrombosis and non-thrombosis in MPA. **sTable 3**. The clinical characteristics between thrombosis and non-thrombosis in GPA. **sTable 4**. The clinical characteristics between thrombosis and non-thrombosis in EGPA.

## Data Availability

No datasets were generated or analysed during the current study.
