# Supplementary material for: Renal damage and old age: risk factors for thrombosis in patients with ANCA-associated vasculitis
Source: Thromb J. 2024 Mar 20;22:29. doi: 10.1186/s12959-024-00593-9 (PMC10953224; doi:10.1186/s12959-024-00593-9)
Supplement: Supplementary file 1 — Supplementary Material 1: sFigure 1. Calibration curves of the predicted nomogram. The x-axis represents the predicted probability calculated by the nomogram, and the y-axis is the observed actual probability of thrombotic events in AAV patients. Results of the Hosmer-Lemeshow test demonstrate that the P-value of 0.541. sFigure 2. The decision curve analysis (DCA) of the nomogram. The horizontal line indicates no AAV patients with thrombosis, and the red diagonal line indicates AAV patients with thrombosis. The solid blue line indicates the risk nomogram. In DCA, the nomogram shows more net benefit THAN full or no treatment across a threshold probability range. sTable 1. The rate of thrombosis in different AAV classifications. sTable 2. The clinical characteristics between thrombosis and non-thrombosis in MPA. sTable 3. The clinical characteristics between thrombosis and non-thrombosis in GPA. sTable 4. The clinical characteristics between thrombosis and non-thrombosis in EGPA. [file 12959_2024_593_MOESM1_ESM.docx]

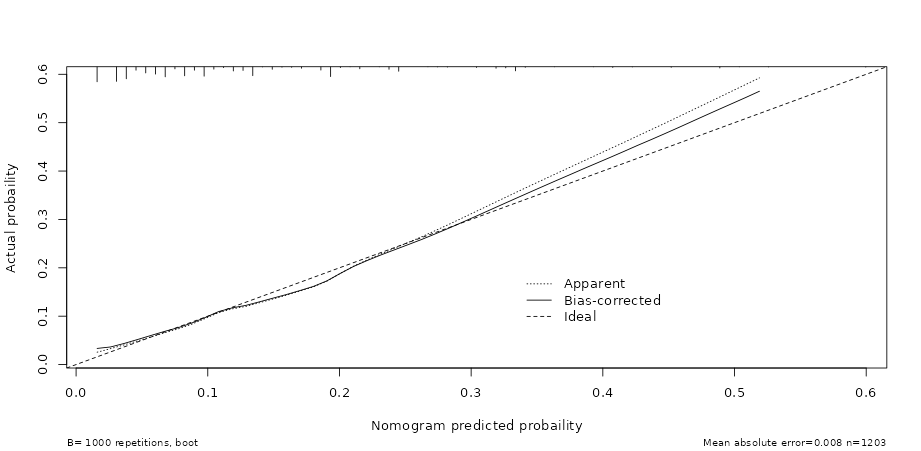
sFigure 1. Calibration curves of the predicted nomogram.

The x-axis represents the predicted probability calculated by the nomogram, and the y-axis is the observed actual probability of thrombosis in AAV patients. Results of the Hosmer-Lemeshow test demonstrate that the P-value of 0.541.


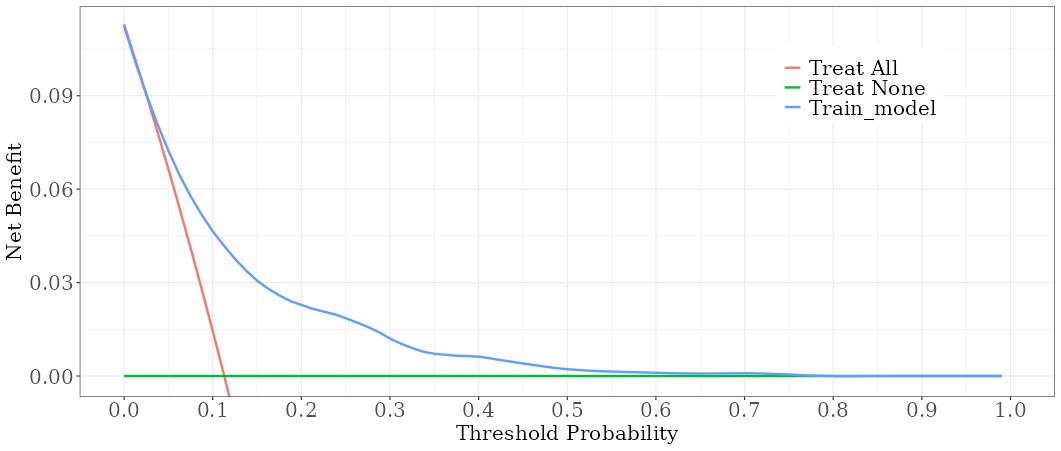
 sFigure 2. The decision curve analysis (DCA) of the nomogram.

The horizontal line indicates no AAV patients with thrombosis, and the red diagonal line indicates AAV patients with thrombosis. The solid blue line indicates the risk nomogram. In DCA, the nomogram shows more net benefit THAN full or no treatment across a threshold probability range.

sTable1. The rate of thrombosis in different AAV classifications.

|  | MPA | GPA | EGPA | P value |
| --- | --- | --- | --- | --- |
| VTE | 57（9.5%） | 27（7.6%） | 36（18.9%） | 0.001 |
| DVT | 47（7.8%） | 22（6.2%） | 32（16.8%） | <0.001 |
| PE | 8（1.3%） | 4（1.1%） | 10（5.3%） | 0.004 |
| ATE | 4（0.7%） | 0（0%） | 4（2.1%） | 0.026 |

* VTE, venous thrombotic embolism; DVT, deep venous thrombosis; PE, pulmonary embolism; ATE, arterial thromboembolism.

sTable 2. The clinical characteristics between thrombosis and non-thrombosis in MPA.

| Variables | Total  (n = 602) | Thrombosis  (n = 63) | Non-thrombosis  (n = 539) | P value |
| --- | --- | --- | --- | --- |
| Age, Mean ± SD | 60.62 ± 14.92 | 66.54 ± 9.79 | 59.93 ± 15.26 | <.001 |
| Gender (Male), n(%) | 281 (46.68) | 23 (36.51) | 258 (47.87) | 0.087 |
| System involvement, n(%) | | |  |  |
| Kidney | 507 (84.22) | 56 (88.89) | 451 (83.67) | 0.283 |
| Lung | 385 (63.95) | 47 (74.60) | 338 (62.71) | 0.063 |
| Neurological | 86 (14.29) | 9 (14.29) | 77 (14.29) | 1.000 |
| ENT | 84 (13.95) | 14 (22.22) | 70 (12.99) | 0.045 |
| Commodities, n(%) | |  |  |  |
| Hypertension | 329 (54.65) | 43 (68.25) | 286 (53.06) | 0.022 |
| Diabetes | 195 (32.39) | 17 (26.98) | 178 (33.02) | 0.332 |
| CAD | 48 (7.97) | 8 (12.70) | 40 (7.42) | 0.143 |
| CHD | 35 (5.81) | 3 (4.76) | 32 (5.94) | 0.926 |
| CKD | 146 (24.25) | 23 (36.51) | 123 (22.82) | 0.016 |
| ESRD | 115 (19.10) | 22 (34.92) | 93 (17.25) | <.001 |
| COPD | 22 (3.65) | 2 (3.17) | 20 (3.71) | 1.000 |
| Stroke | 52 (8.64) | 7 (11.11) | 45 (8.35) | 0.460 |
| Hypoalbuminemia, n(%) | 182 (31.06) | 25 (40.98) | 157 (29.90) | 0.077 |
| Scr (μmol/L), n(%) | |  |  | <.001 |
| <124 | 251 (42.83) | 16 (26.23) | 235 (44.76) |  |
| 125-249 | 136 (23.21) | 13 (21.31) | 123 (23.43) |  |
| 249-499 | 119 (20.31) | 13 (21.31) | 106 (20.19) |  |
| >500 | 80 (13.65) | 19 (31.15) | 61 (11.62) |  |
| D-dimer (mg/L), M (Q₁, Q₃) | 1.75 (0.77, 4.14) | 4.25 (1.44, 8.36) | 1.56 (0.69, 3.63) | <.001 |
| Elevated ESR, n(%) | 411 (79.96) | 42 (84.00) | 369 (79.53) | 0.453 |
| Elevated CRP, n(%) | 318 (57.71) | 42 (70.00) | 276 (56.21) | 0.041 |
| Decreased C3, n(%) | 76 (17.92) | 11 (25.58) | 65 (17.06) | 0.167 |
| Decreased C4, n(%) | 38 (8.82) | 6 (13.95) | 32 (8.25) | 0.333 |
| Complications, n(%) | |  |  |  |
| Infection | 182 (30.23) | 23 (36.51) | 159 (29.50) | 0.252 |
| Anemia | 257 (42.69) | 36 (57.14) | 221 (41.00) | 0.014 |
| Treatment, n(%) | |  |  |  |
| GC po. | 584 (97.01) | 61 (96.83) | 523 (97.03) | 1.000 |
| GC pulse. | 158 (26.25) | 20 (31.75) | 138 (25.60) | 0.294 |
| CTX | 447 (74.25) | 45 (71.43) | 402 (74.58) | 0.588 |
| RTX | 28 (4.65) | 3 (4.76) | 25 (4.64) | 1.000 |
| PE | 30 (4.98) | 5 (7.94) | 25 (4.64) | 0.405 |
| Death, n(%) | 41 (6.81) | 4 (6.35) | 37 (6.86) | 1.000 |
| Length of stay, Mean ± SD | 22.33 ± 13.04 | 26.65 ± 15.78 | 21.82 ± 12.60 | 0.005 |

sTable 3. The clinical characteristics between thrombosis and non-thrombosis in GPA.

| Variables | Total  (n = 357) | Thrombosis  (n = 31) | Non-thrombosis  (n = 326) | P value |
| --- | --- | --- | --- | --- |
| Age, Mean ± SD | 47.66 ± 16.27 | 52.19 ± 12.32 | 47.23 ± 16.55 | 0.045 |
| Gender (Male), n(%) | 196 (54.90) | 21 (67.74) | 175 (53.68) | 0.133 |
| System involvement, n(%) | | |  |  |
| Kidney | 184 (51.54) | 19 (61.29) | 165 (50.61) | 0.256 |
| Lung | 170 (47.62) | 19 (61.29) | 151 (46.32) | 0.111 |
| Neurological | 60 (16.81) | 15 (48.39) | 45 (13.80) | <.001 |
| ENT | 164 (45.94) | 16 (51.61) | 148 (45.40) | 0.507 |
| Commodities, n(%) | |  |  |  |
| Hypertension | 122 (34.17) | 14 (45.16) | 108 (33.13) | 0.177 |
| Diabetes | 91 (25.49) | 6 (19.35) | 85 (26.07) | 0.412 |
| CAD | 18 (5.04) | 2 (6.45) | 16 (4.91) | 1.000 |
| CHD | 6 (1.68) | 1 (3.23) | 5 (1.53) | 0.423 |
| CKD | 34 (9.52) | 3 (9.68) | 31 (9.51) | 1.000 |
| ESRD | 20 (5.60) | 4 (12.90) | 16 (4.91) | 0.150 |
| COPD | 5 (1.40) | 0 (0.00) | 5 (1.53) | 1.000 |
| Stroke | 22 (6.16) | 4 (12.90) | 18 (5.52) | 0.214 |
| Hypoalbuminemia, n(%) | 77 (22.19) | 10 (34.48) | 67 (21.07) | 0.096 |
| Scr (μmol/L), n(%) |  |  |  | 0.063 |
| <124 | 275 (79.25) | 19 (65.52) | 256 (80.50) |  |
| 125-249 | 37 (10.66) | 4 (13.79) | 33 (10.38) |  |
| 249-499 | 24 (6.92) | 3 (10.34) | 21 (6.60) |  |
| >500 | 11 (3.17) | 3 (10.34) | 8 (2.52) |  |
| D-dimer (mg/L), M (Q₁, Q₃) | 1.13 (0.38, 2.94) | 3.46 (1.45, 7.59) | 1.04 (0.38, 2.58) | <.001 |
| Elevated ESR, n(%) | 233 (72.14) | 16 (59.26) | 217 (73.31) | 0.119 |
| Elevated CRP, n(%) | 207 (63.50) | 13 (50.00) | 194 (64.67) | 0.136 |
| Decreased C3, n(%) | 22 (8.94) | 3 (13.04) | 19 (8.52) | 0.734 |
| Decreased C4, n(%) | 14 (5.69) | 3 (13.04) | 11 (4.93) | 0.260 |
| Complications, n(%) | |  |  |  |
| Infection | 86 (24.09) | 13 (41.94) | 73 (22.39) | 0.015 |
| Anemia | 75 (21.01) | 9 (29.03) | 66 (20.25) | 0.251 |
| Treatment, n(%) | |  |  |  |
| GC po. | 345 (96.64) | 31 (100.00) | 314 (96.32) | 0.572 |
| GC pulse. | 83 (23.25) | 11 (35.48) | 72 (22.09) | 0.092 |
| CTX | 262 (73.39) | 25 (80.65) | 237 (72.70) | 0.339 |
| RTX | 39 (10.92) | 3 (9.68) | 36 (11.04) | 1.000 |
| PE | 6 (1.68) | 2 (6.45) | 4 (1.23) | 0.088 |
| Death, n(%) | 13 (3.64) | 2 (6.45) | 11 (3.37) | 0.710 |
| Length of stay, Mean ± SD | 25.06 ± 15.03 | 36.71 ± 19.63 | 23.95 ± 14.07 | 0.001 |

sTable 4. The clinical characteristics between thrombosis and non-thrombosis in EGPA.

| Variables | Total  (n = 190) | Thrombosis  (n = 37) | Non-thrombosis  (n = 153) | P value |
| --- | --- | --- | --- | --- |
| Age, Mean ± SD | 48.58 ± 16.11 | 56.78 ± 15.08 | 46.59 ± 15.76 | <.001 |
| Gender (Male), n(%) | 114 (60.00) | 23 (62.16) | 91 (59.48) | 0.765 |
| System involvement, n(%) | | |  |  |
| Kidney | 56 (29.47) | 13 (35.14) | 43 (28.10) | 0.400 |
| Lung | 78 (41.05) | 26 (70.27) | 52 (33.99) | <.001 |
| Neurological | 67 (35.26) | 17 (45.95) | 50 (32.68) | 0.130 |
| ENT | 65 (34.21) | 11 (29.73) | 54 (35.29) | 0.522 |
| Commodities, n(%) | |  |  |  |
| Hypertension | 54 (28.42) | 14 (37.84) | 40 (26.14) | 0.157 |
| Diabetes | 40 (21.05) | 9 (24.32) | 31 (20.26) | 0.586 |
| CAD | 13 (6.84) | 5 (13.51) | 8 (5.23) | 0.153 |
| CHD | 7 (3.68) | 3 (8.11) | 4 (2.61) | 0.269 |
| CKD | 8 (4.21) | 3 (8.11) | 5 (3.27) | 0.390 |
| ESRD | 6 (3.16) | 4 (10.81) | 2 (1.31) | 0.015 |
| COPD | 11 (5.79) | 4 (10.81) | 7 (4.58) | 0.287 |
| Stroke | 16 (8.42) | 5 (13.51) | 11 (7.19) | 0.361 |
| Hypoalbuminemia, n(%) | 29 (15.93) | 8 (21.62) | 21 (14.48) | 0.290 |
| Scr (μmol/L), n(%) |  |  |  | 0.151 |
| <124 | 166 (91.21) | 31 (83.78) | 135 (93.10) |  |
| 125-249 | 7 (3.85) | 2 (5.41) | 5 (3.45) |  |
| 249-499 | 7 (3.85) | 3 (8.11) | 4 (2.76) |  |
| >500 | 2 (1.10) | 1 (2.70) | 1 (0.69) |  |
| D-dimer (mg/L), M (Q₁, Q₃) | 1.02 (0.38, 2.58) | 2.59 (1.02, 5.89) | 0.79 (0.32, 1.67) | <.001 |
| Elevated ESR, n(%) | 85 (50.00) | 16 (45.71) | 69 (51.11) | 0.569 |
| Elevated CRP, n(%) | 77 (44.77) | 18 (52.94) | 59 (42.75) | 0.285 |
| Decreased C3, n(%) | 6 (4.72) | 1 (4.00) | 5 (4.90) | 1.000 |
| Decreased C4, n(%) | 5 (3.91) | 1 (4.00) | 4 (3.88) | 1.000 |
| Complications, n(%) | |  |  |  |
| Infection | 41 (21.58) | 10 (27.03) | 31 (20.26) | 0.369 |
| Anemia | 32 (16.84) | 9 (24.32) | 23 (15.03) | 0.175 |
| Treatment, n(%) | |  |  |  |
| GC po. | 182 (95.79) | 36 (97.30) | 146 (95.42) | 0.958 |
| GC pulse. | 44 (23.16) | 7 (18.92) | 37 (24.18) | 0.496 |
| CTX | 132 (69.47) | 30 (81.08) | 102 (66.67) | 0.088 |
| RTX | 6 (3.16) | 0 (0.00) | 6 (3.92) | 0.484 |
| PE | 1 (0.53) | 0 (0.00) | 1 (0.65) | 1.000 |
| Death, n(%) | 3 (1.58) | 0 (0.00) | 3 (1.96) | 1.000 |
| Length of stay, Mean ± SD | 23.68 ± 13.59 | 28.68 ± 15.40 | 22.47 ± 12.88 | 0.012 |
